# INTERNET USE, PERCEIVED CONTROL, AND INFORMATION ANXIETY AMONG CHINESE OLDER ADULTS

**DOI:** 10.1093/geroni/igae098.4229

**Published:** 2024-12-31

**Authors:** Jiaan Zhang

**Affiliations:** Fudan University, Fudan University, Shanghai, China (People’s Republic)

## Abstract

Despite increasing evidence that Internet use may contribute to Internet-related anxiety in the digital age, studies specifically focusing on older adults remain limited. This study investigates the association between Internet use and information anxiety among older adults, and examines whether this relationship is mediated by perceived control. We analyzed newly collected data from a random sample of adults aged 55 and older living in Shanghai, China in 2024. The total sample comprised 428 older Internet users. Internet use was assessed by measuring the duration of Internet use, frequency of Internet use, and types of Internet use. Information anxiety was measured using a 23-item scale based on Wurman’s theory of Information Anxiety, with each item rated on a 5-point scale ranging from strongly disagree to strongly agree. Perceived control was evaluated by asking respondents to indicate their agreement with statements including four items of personal mastery and eight items of perceived constraints, using a 7-point scale. The results indicate that the duration of Internet use is significantly associated with lower levels of information anxiety, and that Internet use for communication and social use reduces information anxiety. Additionally, perceived control is negatively associated with information anxiety. Further analysis reveals that perceived control partially mediates the relationship between Internet use duration and information anxiety. These findings have important implications for developing effective interventions to alleviate Internet-related anxiety among older adults.

